# Immune characterization of metastatic colorectal cancer patients post reovirus administration

**DOI:** 10.1186/s12885-020-07038-2

**Published:** 2020-06-18

**Authors:** Ruwan Parakrama, Elisha Fogel, Carol Chandy, Titto Augustine, Matt Coffey, Lydia Tesfa, Sanjay Goel, Radhashree Maitra

**Affiliations:** 1 Montefiore Medical Center, 1695 Eastchester Road, Bronx, NY 10461 USA; 2 Department of Biology, Yeshiva University, 500 West W 185th Street, New York, NY 10033 USA; 3 Albert Einstein College of Medicine, 1300 Morris Park Ave, Bronx, NY 10461 USA; 4 Oncolytics Biotech, Calgary, Canada

**Keywords:** Reovirus, Colorectal cancer, Immune profile, CD8 +, *KRAS*, *VEGFA*

## Abstract

**Background:**

*KRAS* mutations are prevalent in 40–45% of patients with colorectal cancer (CRC) and targeting this gene has remained elusive. Viruses are well known immune sensitizing agents. The therapeutic efficacy of oncolytic reovirus in combination with chemotherapy is examined in a phase 1 study of metastatic CRC. This study evaluates the nature of immune response by determining the cytokine expression pattern in peripheral circulation along with the distribution of antigen presenting cells (APCs) and activated T lymphocytes. Further the study evaluates the alterations in exosomal and cellular microRNA levels along with the effect of reovirus on leukocyte transcriptome.

**Methods:**

Reovirus was administered as a 60-min intravenous infusion for 5 consecutive days every 28 days, at a tissue culture infective dose (TCID_50_) of 3 × 10^10^. Peripheral blood mononuclear cells (PBMC) were isolated from whole blood prior to reovirus administration and post-reovirus on days 2, 8, and 15. The expression profile of 25 cytokines in plasma was assessed (post PBMC isolation) on an EMD Millipore multiplex Luminex platform. Exosome and cellular levels of miR-29a-3p was determined in pre and post reovirus treated samples. Peripheral blood mononuclear cells were stained with fluorophore labelled antibodies against CD4, CD8, CD56, CD70, and CD123, fixed and evaluated by flow cytometry. The expression of granzyme B was determined on core biopsy of one patient. Finally, Clariom D Assay was used to determine the expression of 847 immune-related genes when compared to pre reovirus treatment by RNA sequencing analysis. A change was considered if the expression level either doubled or halved and the significance was determined at a *p* value of 0.001.

**Results:**

Cytokine assay indicated upregulation at day 8 for IL-12p40 (2.95; *p* = 0.05); day 15 for GM-CSF (3.56; *p* = 0.009), IFN-y (1.86; *p* = 0.0004) and IL-12p70 (2.42; *p* = 0.02). An overall reduction in IL-8, VEGF and RANTES/CCL5 was observed over the 15-day period. Statistically significant reductions were observed at Day 15 for IL-8 (0.457-fold, 53.3% reduction; *p* = 0.03) and RANTES/CC5 (0.524-fold, 47.6% reduction; *p* = 0.003). An overall increase in IL-6 was observed, with statistical significance at day 8 (1.98- fold; 98% increase, *p* = 0.00007). APCs were stimulated within 48 h and activated (CD8^+^ CD70^+^) T cells within 168 h as determine by flow cytometry. Sustained reductions in exosomal and cellular levels of miR-29a-3p (a microRNA upregulated in CRC and associated with decreased expression of the tumor suppressor *WWOX* gene) was documented. Reovirus administration further resulted in increases in *KRAS* (33x)*, IFNAR1* (20x), *STAT3*(5x), and *TAP1* (4x) genes after 2 days; *FGCR2A* (23x) and *CD244* (3x) after 8 days; *KLRD1* (14x), *TAP1* (2x) and *CD244*(2x) after 15 days. Reductions (> 0.5x) were observed in *VEGFA* (2x) after 2 days*; CXCR2* (2x), *ITGAM* (3x) after 15 days.

**Conclusions:**

Reovirus has profound immunomodulatory properties that span the genomic, protein and immune cell distribution levels. This is the first study with reovirus in cancer patients that demonstrates these multi- layered effects, demonstrating how reovirus can function as an immune stimulant (augmenting the efficacy of immuno-chemo-therapeutic drugs), and an oncolytic agent. Reovirus thus functions bimodally as an oncolytic agent causing lysis of tumor cells, and facilitator of immune-mediated recognition and destruction of tumor cells.

**Graphical abstract:**

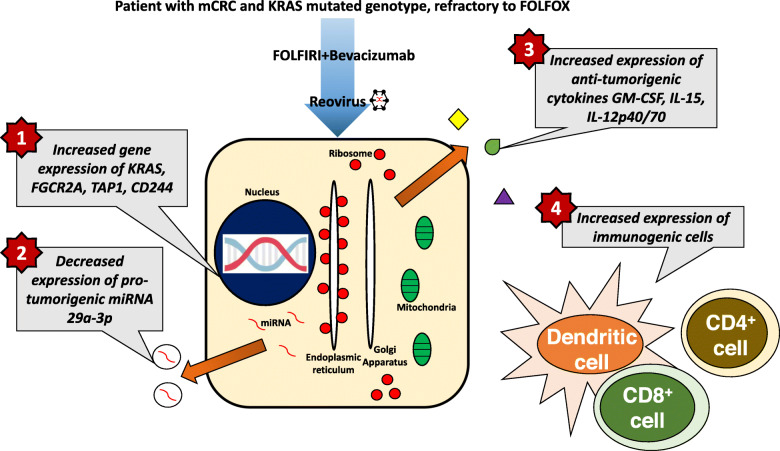

## Background

Colorectal cancer (CRC) is the third most common cancer in men and women in the US and is the second leading cause of cancer-related death [1]. In spite of major advances, the 5-year survival rate for patients with metastatic disease remains at 15% [2]. Pharmacotherapy for mCRC has evolved over time, from the initial regimen of 5-FU/Leucovorin to the current standard of care of FOLFOX or FOLFIRI, with anti-VEGF (e.g., bevacizumab), anti-EGFR (e.g., cetuximab) molecules also being integrated into treatment protocols [3]. Additionally, the approvals in 2017 of PD-1 immune checkpoint inhibitors nivolumab and pembrolizumab for patients with a sub-type of CRC (mismatch repair deficient, dMMR) [4] has further expanded the therapeutic armamentarium.

However, certain patients do not respond as robustly (or at all) to the aforementioned biologic therapies and it has been hypothesized that this is a result of differences within the tumor microenvironment (TME). Immune checkpoint inhibitors, for example work best when there is a large lymphocytic infiltrate within the TME, which is significantly decreased in CRC [5]. In a sub-group of CRC patients, activating mutations in the KRAS gene further prevents utilization of anti-EGFR therapies, as they act upstream of the mutated pathway [6].

It is therefore clear that there is a need for alternative adjuvant compounds that can either bypass compromised molecular pathways or restore the TME to allow recognition, activation and destruction of cancer cells by the immune system. Oncolytic viruses may provide the latter, as their infection into host cells results in increased levels of local cytokine expression and an influx of immune cells including natural killer (NK) cells, activated T cells and antigen-presenting cells (APCs) [5].

Reovirus (a naturally occurring, ubiquitous double stranded (ds) RNA virus) has been shown to preferentially replicate in and be cytopathic to transformed cells possessing an activated *KRAS*-signaling pathway [7], demonstrate in vivo activity in CRC cell-line models [8], and have synergistic activity with irinotecan in KRAS-mutated CRC cell lines and xenograft models [9–11]. In the current paper, we examined the immunomodulatory effects of reovirus across genomic, protein and immune cell distribution levels, as part of a phase 1 clinical study of mCRC patients with KRAS mutations receiving FOLFIRI and bevacizumab treatment.

Specifically, we studied (i.e., following virus administration) the genomic effects of reovirus by examining the comprehensive modulation of gene expression over 15 days’ time by transcriptome analysis, and the expression of selected microRNA (miRNA) in the exosomal and cellular RNA by RT- PCR. Effects on protein signaling were studied via the release of serum cytokines using an ELISA method. Lastly, effects on populations of immune cells were examined via analysis of cell surface markers on dendritic cells, T lymphocytes and natural killer cells by flow cytometry. We demonstrate that administration of reovirus results in several gene expression changes, an increase in the levels of anti- tumorigenic cytokines, a reduction in pro-tumorigenic cytokines and miRNA, and an increase in the populations of APCs and activated T cells. The potential interplay (i.e., between genes, cytokines and immune cells) across these changes, including in the context of preliminary clinical data from the clinical trial is discussed.

## Methods

### Ethical considerations

The entire study was performed in compliance with Institutional and Federal guidelines for clinical research. All patient tissue and blood samples were drawn with informed consent based on a local IRB approved consent form.

### Serum sample collection

Serum samples were obtained from 8 patients, all having KRAS-mutated mCRC. Five (5) patients had received reovirus as part of a phase 1 clinical trial (NCT01274624; 11). Three (3) patients were not enrolled in the trial but did receive equivalent background chemotherapy (i.e., FOLFIRI and bevacizumab).

### Reovirus administration

Reovirus was supplied by Oncolytics Biotech, Inc. as a translucent to clear, colorless to light blue liquid in vials containing 7.2 × 10^11^ tissue culture infective dose (TCID_50_) per ml of reovirus in a phosphate- buffered solution and stored at minus 70 °C. Reovirus was administered as a 60-min infusion for 5 consecutive days every 28 days, at a tissue culture infective dose (TCID_50_) of 3 × 10^10^/day. Plasma was collected pre-reovirus, at 24 and 48 h, and 7 and 14 days after first dose of reovirus.

### Flow cytometry

To assess the immune-modulating effects of reovirus, blood was drawn into cell preparation tubes (CPT) (BD Vacutainer® CPT™, Mononuclear Cell Preparation Tubes, (manufacturer # 362753) to isolate peripheral blood mononuclear cells (PBMC). Fluorescence activated cell sorting (FACS) assay was performed using fluorophore labeled antibody staining for T helper lymphocyte (FITC-CD4; catalog # 11–0049; Thermofisher-eBiosciences), cytotoxic T lymphocyte (PE-CD8; catalog # 12–0088; Thermofisher-eBiosciences), activated cytotoxic T lymphocyte (CD70-eFluor 660; catalog # 50–0709; Thermofisher-eBiosciences), mature dendritic cell (CD123-PE-Cy7; catalog # 25–1239; Thermofisher- eBiosciences) and Natural killer (NK) cells (CD56-eFluor 450 catalog # 48–0566; Thermofisher- eBiosciences) along with live dead marker (FVD-eFluor 780; catalog # 65–0865 Thermofisher- eBiosciences). The staining and data acquisition was performed within 3 h of sample collection. Flo Jo software (version 9.8.1) was used for all analysis and gating was maintained unaltered throughout the entire analysis.

### ELISA

Cytokine assay – serum samples (post PBMC isolation) from the 5 patients who received reovirus were centrifuged for 30 min at 12,500 G. Next, 50 μL of supernatant was added to a 96-well plate (samples were added in triplicate). EMD Millipore beads coated with the following cytokines were then added to each sample (Cat#HCYTOMAG-60K-25): IL-1 alpha, IL-1 beta, IL-2, IL-3, IL-4, IL-6, IL-7, IL-8, IL-9, IL-10, IL-12 (p40), IL-12 (p70), IL-13, IL-15, IL-17A, GM-CSF, IFN-alpha 2, IFN gamma, TNF alpha, MCP-1, MCP-3, MIP-1 alpha, MIP-1 beta, RANTES and VEGF. Cytokine expression profiles were assessed on an EMD Millipore multiplex Luminex platform. Data from one patient who received reovirus is not included in this paper, as the majority of the Luminex output across all cytokines was below the limit of detection. All data were normalized to respective pre-reovirus administration for each patient to serve as controls for the study.

Additionally the data from the 3 patients who did not receive reovirus were run through similar calculations to confirm that there was no change in cytokine expression over time. Thus the observed alterations in cytokine expression can be related to reovirus administration and not due to FOLFIRI and bevacizumab administration (Data not shown).

### Immunohistochemistry

Biopsied specimens (from 1 patient who received reovirus: resected colostomy for pre reovirus sample; core biopsy for post reovirus sample) were fixed in neutral buffered formalin and subjected to paraffin embedding. The 5-μm-thick 10% formalin-fixed, paraffin-embedded tissue sections were deparaffinized in xylene three times, 10 min each, and subsequently rehydrated through graded alcohols to distilled water. Antigen heat retrieval was performed in 1 mM EDTA (pH 9.0) for 10 min (PMS2 15 min) using a microwave oven. Next, the sections were allowed to cool down in room temperature for 1.5 h. After rinsing in distilled water and TBS successively, sections were incubated with rabbit polyclonal Granzyme B (Thermofisher Scientific # PA5-13518) at 4 °C for 2 h [11]. Finally, the sections were covered with streptavidin peroxidase (Dako, Santa Barbara, CA) diluted 1:100 in PBS, incubated for 30 min at 37°C, washed three times in PBS and stained with 3,3’-diaminobenzidine as a substrate for the peroxidase for approximately 30 min at 37°C. Counter staining was performed using Mayer’s hematoxylin. Slides were scanned on the 3DHistech P250 slide scanner (SIG #1S10OD019961-01) using a 20X objective. Brown stain analysis was completed on the whole piece of tissue on every slide with 3DHistech Quant Center, using the DensitoQuant module. In this module, brown stain pixels were distinguished from the rest of the tissue by color thresh-holding. The analysis of blue versus brown areas of tissue was completed in ImageJ, using the color threshold module. Finally, cells were viewed under 40X magnification in Case Viewer 2.3.

### Micro RNA analysis

For exosomal miRNA analysis, serum samples (from the 5 patients treated with reovirus and the 3 patients treated with background therapy only) were centrifuged at 12,500 G for 30 min. miRNA from 200 μL of supernatant was then extracted using Qiagen’s miRNeasy Serum/Plasma Advanced Kit (Cat#217204). For cellular miRNA analysis, PBMC cell pellets were lysed, and miRNA was extracted using Qiagen’s RNEasy FFPE kit (Cat#74404). 10 μg of total miRNA were analyzed (in quadruplicate for each sample) using miRNA assays 29a-3p, 26a-5p, 21-5p, 99-5p and 337-3p, obtained from ThermoFisher Scientific. RT-PCR quantification of miRNA assays for all samples was done using Applied Biosystems Taqman Advanced miRNA cDNA synthesis kit (Cat#A28007), with Cq analysis performed on Bio-RadCFX96 real time system C1000 touch thermal cycle.

### Transcriptome analysis

Following total RNA isolation (from the 5 patients treated with reovirus and the 3 patients treated with background therapy only), single stranded cDNA synthesis was prepared by the method of fragmentation and labeling (ThermoFisher Scientific, Clariom D Pico Assay, human with arrays [Catalog Number 902924]). Briefly, total RNA was reverse transcribed using a reverse transcription priming method from an engineered set of primers that exclude sequences that match ribosomal RNA (rRNA). Thus, non- ribosomal RNA from the sample was primed (including both poly(A) and non-poly(A) mRNA) and converted into double-stranded cDNA using first and second strand enzymes from the said kit. Templates were used for in vitro transcription reactions at 37 °C for 16 h to yield cRNA. Next, hybridization cocktail from the hybridization kit (Catalog Number 900454) was used to generate ss-cDNA from cRNA by the technique of chemical fragmentation followed by biotin labeling. Strictly adhering to the Affymetrix Genechip protocol, the ss-cDNA was hybridized to the Clariom D GeneChip probe array. The array image was finally generated by a high-resolution GeneArray Scanner 3000 7G (ThermoFisher Scientific, Santa Clara, CA,). For this study we analyzed 847 immune related genes (Supplementary Table 1) and compared the post reovirus treatment at days 2, 8 and 15 to the pre-reovirus treatment samples. Up or down regulation was reported for all significant changes (*p* < 0.001) in expression, and those with a two- fold change in upregulation or half-fold change in down regulation were further analyzed.

### Statistical methods

For the transcriptome analysis, individual sample signals for each patient at each time point were extracted from the TAC (Transcriptome Assay Console: Thermofisher Scientific) software, organized and compiled in Microsoft Office Excel. Gene expression data were analyzed by the 2−ΔΔCT method [12] and normalized to the individual pre-reovirus (i.e., baseline) levels for each gene. Two tailed t-test was used to determine statistical significance (*p* < 0.001). Statistics were calculated using Excel. For the miRNA and cytokine expression results, all patient samples for a given timepoint were pooled; baseline values were normalized to “1”, and the means for all subsequent timepoints were calculated relative to the baseline value. The standard error of the mean was calculated for each timepoint as well. Statistical significance was determined by using a paired t-test, and values less than or equal to 0.05 were reported.

## Results

### Genomic effects following the administration of reovirus

#### Reduction of miR-29-3p in both exosomal and cellular preparations

**Fig. 1:**
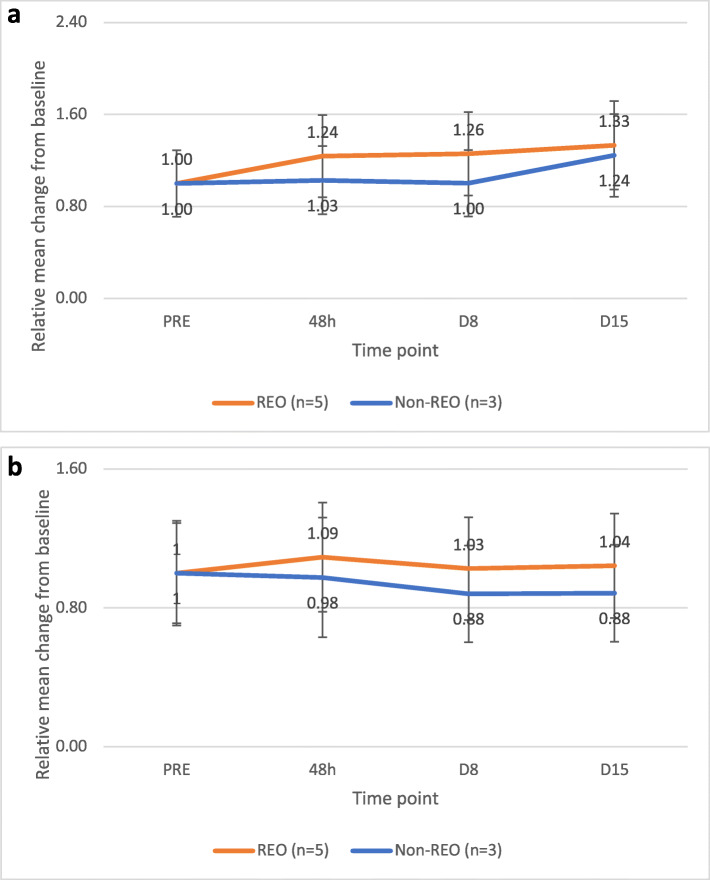
**a** shows the relative change in exosomal miRNA expression (by mean Cq value across all patients) over time. Statistically significant increases in mean Cq (signifying decreases in the quantity of miRNA) were observed at 48 hours (p=0.004), day 8 (p=0.003), and day 15 (p=0.0001) for patients treated with reovirus. Abbreviations: PRE=pre-reovirus administration (baseline); 48h=48 hours; D=Day; REO=patients treated with reovirus; Non-REO=patients treated with background therapy (i.e., bevacizumab and FOLFIRI) only. Error bars represent the standard error of the mean. **b** shows the relative change in cellular miRNA expression (by mean Cq value across all patients) over time. A statistically significant increase in mean Cq (signifying a decrease in the quantity of miRNA) was observed at 48 hours (p=0.002) for patients treated with reovirus. Abbreviations: PRE=prereovirus administration (baseline); 48h=48 hours; D=Day; REO=patients treated with reovirus; Non-REO=patients treated with background therapy (i.e., bevacizumab + FOLFIRI) only Error bars represent the standard error of the mean

In the exosomal samples of patients treated with reovirus, statistically significant *decreases* (indicated by increasing mean Cq values) in the quantity of miR-29-3p relative to pre-reovirus administration were observed for all timepoints (0.0001 <*p* < 0.04; Fig. 1a), compared to patients treated with background therapy only (statistically significant decrease at day 15 only, *p* = 0.001). In the cellular samples, an initial decrease (relative to baseline) in miRNA at 48 h (*p* = 0.002; Fig. 1b) was also observed, however this was not sustained over the days 8 and 15 time points. Patients treated with FOLFIRI and Bevacizumab showed no decrease in miRNA quantity, at any time point.

#### RNA transcriptome analysis

Tables 1 and 2 highlight the expression changes (*p* < 0.001) of various genes at the 48-h, days 8 and 15 timepoints of the REO-022 study. The changes range from increases as high as 33-fold (*KRAS* at 48 h, Table 1), to reductions as low as 3-fold (*ITGAM* at day 15, Table 2). *KRAS, FCGR2A* and *IFNAR1* genes demonstrated ≥20-fold expression increases, following reovirus administration. Fold reductions (Table 2) included *VEGFA* at day 8*, CXCR2* at day 15, *GZMA* at 48 h and day 15, and *ITGAM* at day 15. Additional fold changes (*p* < 0.05) are shown in Supplementary Figure 1a and b.

**Table 1:** Statistically significant fold-increases in gene expression post-reovirus administration (*n* = 5 patients)

Gene	Fold decreases vs. PRE/baseline value; *p* < 0.001		
	48 h	D8	D15
*KRAS*	33		
*FCGR2A*		23	
*IFNAR1*	20		
*STAT3*	5		
*KLRD1*			14
*TAP1*	4		2
*CD244*		3	2

**Table 2:** Statistically significant fold-reductions in gene expression post-reovirus administration (*n* = 5 patients)

	Fold decreases vs. PRE/baseline value; *p* < 0.001		
	48 h	D8	D15
*VEGFA*		2	
*CXCR2*			2
*GZMA*	2		2
*ITGAM*			3

### Protein expression following the administration of reovirus

#### Increases in anti-tumorigenic cytokines and reductions in pro-tumorigenic cytokines

**Fig. 2:**
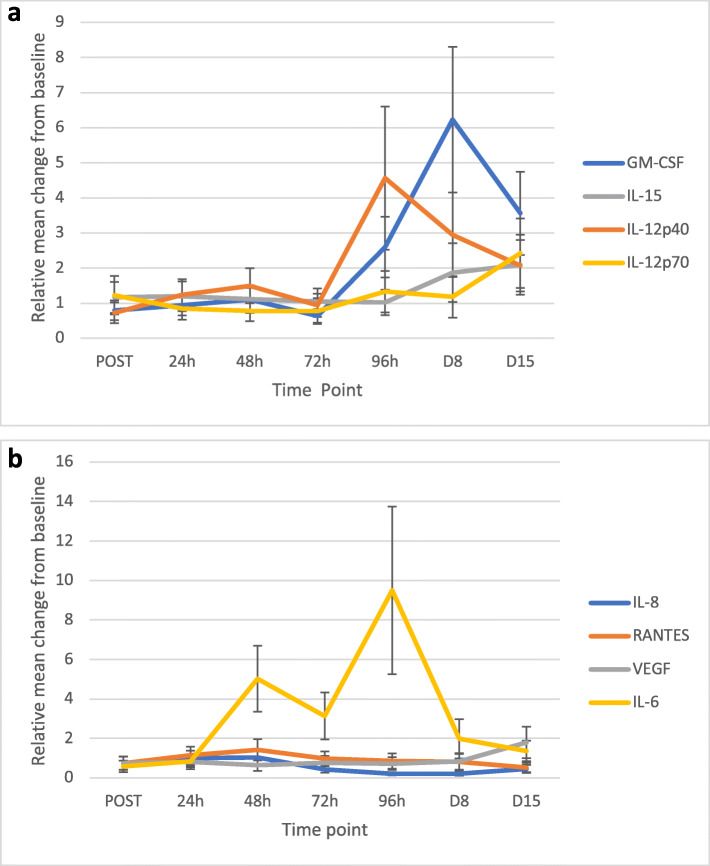
**a** shows the relative change in cytokine expression (by mean expression across all reovirus-treated patients) over time. Statistically significant increases were observed at day 8 for IL-12p40 (p=0.05) and IL-15 (p=0.05); day 15 for GM-CSF (p=0.009) and IL-12p70 (p=0.02). Abbreviations: POST=post-reovirus administration; h=hours; D=Day. Error bars represent the standard error of the mean values. **b** shows the relative change in cytokine expression (by mean expression across all reovirus-treated patients) over time. Statistically significant reductions were observed at 72 hours, 96 hours, day 8 and day 15 for IL-8 (p=0.02; p=0.00003; p=0.00000001; p=0.03); day 15 for RANTES (p=0.003); 24 hours, 48 hours and 72 hours for VEGF (p=0.0002; 0.007; 0.002). Statistically significant increases were observed for the POST and day 8 timepoints for IL-6 (p=0.04; p=0.00007). Abbreviations: POST=post-reovirus administration; h=hours; D=Day. Error bars represent the standard error of the mean values

An overall increase (relative to baseline) in the levels of GM-CSF, IL-15, IL-12p40 and IL-12p70 was observed across the 15-day period (Fig. 2a). Increases were also observed in the levels of IFN-y (Fig. 4) over this period. Statistically significant increases were observed at day 8 for IL-12p40 (2.95; *p* = 0.05); day 15 for GM-CSF (3.56; *p* = 0.009), IFN-y (1.86; *p* = 0.0004) and IL-12p70 (2.42; *p* = 0.02). An overall reduction in IL-8, VEGF and RANTES/CCL5 was observed over the 15-day period (Fig. 2b). Statistically significant reductions (versus baseline values) were observed at Day 15 for IL-8 (0.457 fold, 53.3% reduction; *p* = 0.03) and RANTES/CC5 (0.524 fold, 47.6% reduction; *p* = 0.003). An overall increase in IL-6 was observed, with statistical significance at day 8 (1.98 fold; 98% increase, *p* = 0.00007).

#### Increased granzyme B expression

**Fig. 3:**
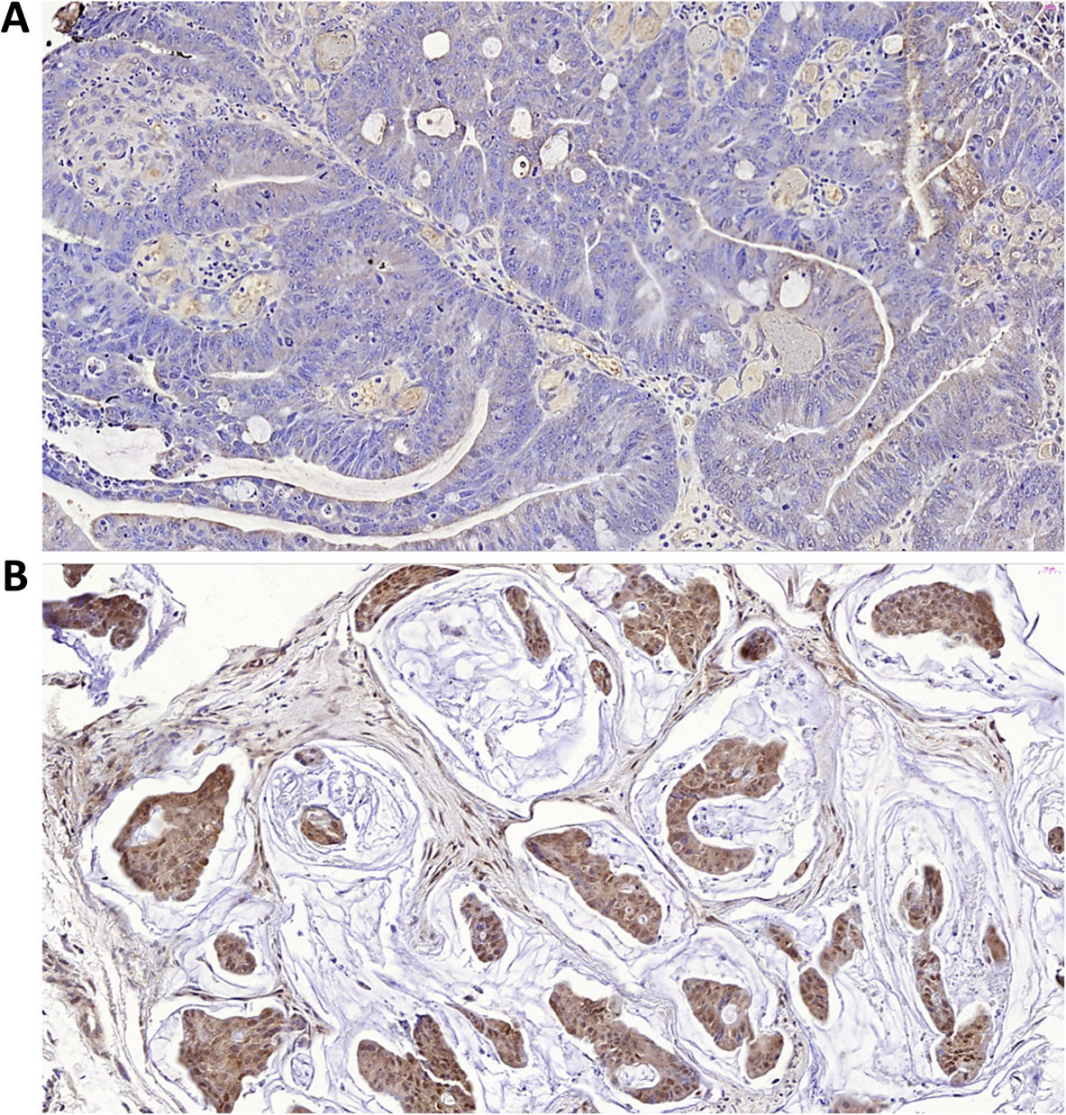
shows pre- (**a**) and post- (**b**) reovirus IHC staining of the biopsied tumor tissue stained with granzyme B at 40X magnification. Post reovirus (b) section shows atrophy of tumor cells in discrete islands with strong staining for granzyme B and surrounded by fibrous connective tissues a phenomenon commonly observed post tumor regression

The relative expression of Granzyme B pre- and post- reovirus treatment was determined by immunohistochemistry. There was a fourfold increase in granzyme B expression in post reovirus treated biopsy (Fig. 3b; core) specimen as compared to initial resection (Fig. 3a) indicating the activation of CD8^+^ T cells upon reovirus administration. The tumor tissues showed significant atrophy with islands of granzyme B positive tumor cell population surrounded by zones of fibrous connective tissue that replaced the dying tumor cells.

### Cellular effects following reovirus administration

#### Increased population of dendritic cells and T lymphocytes

**Fig. 4:**
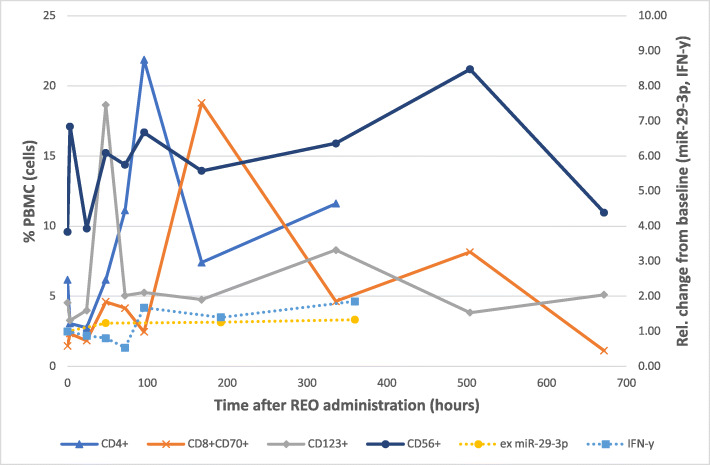
shows the effect of reovirus administration on exosomal miR-29-3p and IFN-y levels, as well as CD4+, CD8+CD70+, CD123+ and CD56+ immune cells. Abbreviations: ex=exosomal; PBMC=peripheral blood mononuclear cells, REO = reovirus

Within 48 h of reovirus administration, a sharp increase in the population of CD123^+^ cells (i.e., dendritic cells) was observed (Fig. 4) for each individual patient, which subsequently resolved to baseline levels. A similar increase in the population of CD8^+^/CD70^+^ cells (i.e., cytotoxic effector T cells) occurred approximately 120 h later (i.e., approximately 168 h following reovirus administration).

The rise in the latter is likely a result of the rise in the former (i.e., presentation of processed reovirus viral particles on the MHC-Class I complex of infected APCs to CD8^+^ T cells, resulting in their activation).

A rise in the population of CD4^+^ T cells was also observed within approximately 96 h of reovirus administration (Fig. 4). The earlier increase (96 vs. 168 h in CD8^+^/CD70^+^ cells) is consistent with the ability of CD4+ cells to activate APCs, providing the co-stimulatory signals to activate additional CD8^+^ effector T cells [13]. The population of CD56^+^ cells (i.e., NK cells) did not change significantly following administration of reovirus.

## Discussion

Oncolytic viruses possess a unique “duality” of action against cancerous cells in comparison with other therapeutic modalities. Not only can they exert cytotoxic effects on cancer cells (via infection, replication and release of viral progeny), but they simultaneously cause activation and proliferation of hitherto dormant immune cells in the TME. This provides a much-needed “jump start” for the immune system, allowing it to recognize and destroy cancer cells, including those which have acquired the ability to thwart host immunity (e.g., through expression of PD-L1, IL-23 and IL-10 receptors resulting in T cell exhaustion [14, 15, 16]).

Reovirus has been shown to preferentially replicate in and be cytopathic to colorectal cancer cells possessing an activated KRAS-signaling pathway [7–10]. In this paper, we have demonstrated that the administration of reovirus against a background of FOLFIRI and bevacizumab therapy in patients with *KRAS*-mutated mCRC results in multiple anti-tumorigenic alterations at the genomic, protein and immune cell distribution levels.

At the genomic level, we showed that reovirus administration results in statistically significant reductions in the exosomal expression of miR-29a-3p, as early as 48 h and sustaining through day 15 (Fig. 1a). This miRNA has been shown to be upregulated in CRC, and is postulated to contribute to CRC pathophysiology via inhibition of the WWOX tumor suppressor gene [17]. It is interesting that in patients treated with background therapy only, a reduction in miR-29a-3p was also seen at day 15. This may be due to suppression from bevacizumab and/or FOLFIRI treatment during the 2-week cycle. Additionally, the difference in the relative mean Cq values at day 15 between patient groups was not statistically significant. However, the sustained reductions in miR-29a-3p following reovirus administration may provide additional benefit via increased IFN-y expression, as miR-29a-3p is known to suppress IFN-y expression in bacterial infections [18]. As shown in Fig. 4, serum increases in IFN-y in patients treated with reovirus were observed after 72 h. These increases were not seen in patients treated with background therapy only (data not shown).

The results of the transcriptome analysis (Tables 1 and 2) highlight additional anti-tumor effects of reovirus. The 4-fold and 2-fold increases at 48 h and at day 15 (respectively) for *TAP1* demonstrate reovirus’ protective effect, as *TAP1* encodes a protein critical for the expression of peptides on the surface of MHC Class I, and down-regulation of this protein has been shown to promote immune evasion and poor prognosis in colorectal cancer [19].

*FCGR2A* and *IFNAR1* genes encode receptors for antibody-binding and Type I interferon-binding, respectively. The observed fold increases in these genes (23-fold at Day 8 for *FCGR2A*; 20-fold at 48 h for *IFNAR1*) are supportive of increased immunogenic activity following reovirus administration, particularly when coupled with the cytokine expression data (Fig. 2), which show increased anti-tumor cytokines (GM-CSF, IL-12p40, IL-12p70 and IL-15 [20]) and decreased pro-tumorigenic cytokines (IL- 8, RANTES, VEGF), over a 15-day period.

Of particular interest in the transcriptome analysis is the 33-fold increase in *KRAS* expression at 48 h (Table 1), and the fold-reductions observed for *VEGFA* (2-fold, day 8), *CXCR2* (2-fold, day 15), *ITGAM* (3-fold, day 15; Table 2). Reovirus infection in normal cells is known to trigger double-stranded RNA activated protein kinase (PKR; inhibits translation of viral proteins) phosphorylation [21]; constitutive expression of KRAS inhibits PKR phosphorylation, explaining the preferential replication of reovirus in *KRAS*-mutated tumor cells. As it is the phosphorylation (and not expression) of PKR that is inhibited by KRAS, the fold increase in *KRAS* seen following reovirus administration may represent an increased feedback inhibition of PKR protein produced in response to reovirus.

The reduction in *VEGFA* (a pro-angiogenic molecule [22]) transcript at day 8 is consistent with the observed reduction of serum VEGF over the preceding time points (Fig. 2b). While the serum reductions are likely due to the effect of bevacizumab, the transcriptome results are due to reovirus, as an examination of the VEGFA expression changes in the patients who did not receive reovirus (but did receive FOLFIRI and bevacizumab), did not show any reduction (data not shown). Furthermore, an additional analysis of genes that are up-regulated by 2-fold and down-regulated by 0.5-fold at a *p*-value < 0.05 showed that *VEGFA* is reduced across 48 h, days 8 and 15 timepoints (Supplementary Figure 1b).

A similar reduction at day 15 was observed for *CXCR2* (the ligand for IL-8, another pro-angiogenic cytokine [23]). Statistically significant reductions in IL-8 were observed across several time points (Fig. 2b). In summary, the reductions in *VEGFA* and *CXCR2* demonstrate anti-tumorigenic effects by reovirus at the genomic level.

Lastly, *ITGAM* encodes CD11b, an integrin which combines with CD18 to form a leukocyte adhesion receptor; bone marrow CD11b+ cells have been shown to promote epithelial-to-mesenchymal transition and metastasis in colorectal cancer [24]. Thus, reductions at day 15 may signify a dampening of metastatic growth of tumor cells by reovirus.

While the aforementioned changes are compelling, it is well known that there is a “tug-of-war” of sorts in the TME [25], between pro- and anti- tumorigenic factors. Thus, the fold increases in STAT3, KLRD1 (CD94) and CD244 (Table 1) also deserve consideration and comment. STAT3 is part of the IL- 6/JAK/STAT3 pathway, which is hyperactive in many cancers and is known to suppress the anti-tumor immune response [26]. The increase in STAT3 is thus likely responsible for the increase observed in serum IL-6 at 24 h post-reovirus administration (Fig. 2b). KLRD1 (CD94) is known to suppress NK activity against tumor cells via ligation with the NKG2A receptor on the tumor cell surface, followed by interaction with HLA-E receptor on the NK cell [27]. As shown in the flow cytometry data in this paper, no change in the population of NK cells was observed after administration of reovirus (Fig. 4). The fold increases seen of CD244 (responsible for NK and T cell exhaustion [28]), at days 8 and 15 may also be contributing to this finding. While it is clear these changes follow reovirus administration, whether they are driven by reovirus in order to support continued viral propagation, versus being a true counter- response by the tumor cell to avoid destruction (following activation of immune cells in the TME by reovirus infection), is beyond the scope of this study and warrants further investigation.

In summary, the data presented in this paper highlight the potential of reovirus to function as an immunomodulatory and cytotoxic adjuvant to standard chemotherapy in patients with mCRC and KRAS- mutations. We have shown that over a 15-day period, reovirus modulates several anti-tumor changes, across the genomic, protein and immune cell distribution levels. Figure 4 presents the temporal and dynamic effects of reovirus between exosomal miR-29-3p, IFN-y and several immune cell types. As mentioned previously, the decrease in miR-29a-3p may contribute to the increase in IFN-y [18]. Additionally, this increase may be attributed to the rising population of activated CD4^+^ and CD8^+^ T-cells, as these predominantly secrete IFN-y [29]. The increased expression of Granzyme B post reovirus treatment in a patient biopsy sample (documented by immunohistochemistry) also demonstrates activation of CD8^+^ T cells, and highlights reovirus-directed tumor cell-specific destruction.

Limitations of this study include a small sample size of patients analyzed who received reovirus (*n* = 5 for all analyses performed, with the exception of the cytokine analysis, in which data from one patient was excluded, and in the immunohistochemistry analysis, in which one patient was biopsied). Additionally, the pooling of patient samples to present (relative) mean changes over time may be biased towards “responder” patients, which the authors have acknowledged by using the standard error of the mean (appropriate in this small sample set to show the variance within the group). However, inter-patient variability across oncologic therapies is not uncommon – indeed, it is one of the main drivers for the discovery of new investigational agents, like reovirus. The results of this study should therefore be appreciated in the context of the known complexity of genomic, protein and cellular interactions within the TME.

## Conclusion

By highlighting the findings above, we encourage the further investigation of reovirus as a therapeutic adjuvant to standard of care therapy, in larger studies which can be appropriately powered to ultimately make definitive statements regarding efficacy and safety. As of the writing of this paper, three out of six patients (50%) who received reovirus had a partial response and the median progression free survival (PFS) and overall survival (OS) were 65.5 and 107.5 weeks, respectively [11]. The PFS and OS results are superior to historic data and the combination treatment was also safe and well tolerated [11]. Thus, administration of reovirus in mCRC patients with KRAS positive mutations represents an important step forward in treatment.

## Supplementary information

**Additional file 1: Supplementary Table 1.** List of immune related genes analyzed by transcriptome assay.

**Additional file 2: Supplementary Figure 1.** a – Transcriptome analysis post-Reovirus administration (Genes up-regulated 2-fold, *p* < 0.05). b – Transcriptome analysis post-Reovirus administration (Genes down-regulated 0.5-fold, *p* < 0.05).

## Data Availability

The datasets used and analyzed during the current study are available from the corresponding author on reasonable request.

## Abbreviations

mCRC: Metastatic colorectal cancer
CD: Cluster of differentiation
APC: Antigen presenting cell
KRAS: Kirsten rat sarcoma
FCGR2A: Fc fragment of Immunoglobulin G receptor IIa
IFNARI: Interferon alpha and beta receptor subunit 1
STAT3: Signal transducer and activator of transcription 3
KLRD1 (CD94): Killer cell lectin-like receptor subfamily D, member 1
TAP1: Transporter 1, ATP binding cassette subfamily B member
CXCR2: C-X-C motif chemokine receptor 2
GZMA: Granzyme A
ITGAM: Integrin subunit alpha M
VEGFA: Vascular endothelial growth factor A
RNA: Ribonucleic acid
PBMC: Peripheral blood mononuclear cells
FOLFIRI: Folinic acid (leucovorin)+fluorouracil (5-FU)+irinotecan
FOLFOX: Folinic acid (leucovorin)+fluorouracil (5-FU)+oxaliplatin
miR: microRNA
WWOX: WW domain-containing oxidoreductase
GM-CSF: Granulocyte-macrophage colony-stimulating factor
TNF: Tumor necrosis factor
IL: Interleukin
IFN: Interferon
MCP: Membrane cofactor protein
MIP: Major intrinsic protein
RANTES (CCL5): Regulated upon activation, normally T cell expressed and presumably secreted
TME: Tumor microenvironment
dMMR: Mismatch repair deficient
EGFR: Epidermal growth factor receptor
NK: Natural killer
RT-PCR: Real-time polymerase chain reaction
ELISA: Enzyme-linked immunoabsorbent assay
IRB: Institutional review board
TCID50: Tissue culture infective dose
FACS: Fluorescence activated cell sorting
FITC-CD4: Fluorophore labeled antibody staining for T helper lymphocyte
