# Supplementary material for: Immune characterization of metastatic colorectal cancer patients post reovirus administration
Source: BMC Cancer. 2020 Jun 18;20:569. doi: 10.1186/s12885-020-07038-2 (PMC7301987; doi:10.1186/s12885-020-07038-2)
Supplement: Supplementary file 1 — Additional file 1: Supplementary Table 1. List of immune related genes analyzed by transcriptome assay. [file 12885_2020_7038_MOESM1_ESM.pdf]

# Supplementary Table 1: List of immune related genes analyzed by transcriptome assay.

| Gene Symbol | | | | | | | | | | | | | | | | | | | | | | | | | | | | | | | | | |
|---|---|---|---|---|---|---|---|---|---|---|---|---|---|---|---|---|---|---|---|---|---|---|---|---|---|---|---|---|---|---|---|---|---|
| 1 | S100A8 | 51 | MAPKAPK | 101 | CD44 | 151 | ISG15 | 201 | HCK | 251 | FCGR3A | 301 | TLR6 | 351 | CD24 | 401 | DDX50 | 451 | IL26 | 501 | CD3EAP | 551 | CLEC4A | 601 | IRF3 | 651 | PTGDR2 | 701 | NOD2 | 751 | CTCFL | 801 | TPSAB1; |
| 2 | ITGB1 | 52 | CLU; MIR6 | 102 | GNLY | 152 | LY86 | 202 | ICOSLG | 252 | IL1RN | 302 | IKBKG | 352 | CD38 | 402 | CTAG1B; C | 452 | IDO1 | 502 | MAGEC2 | 552 | IGF2R | 602 | TNFRSF13 | 652 | IL16 | 702 | IL3RA | 752 | SMPD3 | 802 | TNFRSF18 |
| 3 | FCER1G | 53 | TGFB1 | 103 | NCF4 | 153 | ETS1 | 203 | GZMA | 253 | CR2 | 303 | IL10 | 353 | MSR1 | 403 | TMED7-TI | 453 | TOLLIP | 503 | MPPED1 | 553 | LTA | 603 | CSF3R | 653 | DDX43 | 703 | TNFRSF9 | 753 | CD1A | 803 | LAG3 |
| 4 | RPS6 | 54 | EDC3 | 104 | IFI35 | 154 | C6 | 204 | SPA17 | 254 | PMCH | 304 | KLRG1 | 354 | CXCL13 | 404 | BLK | 454 | GAGE1 | 504 | SMAD3 | 554 | CCRL2 | 604 | XCR1 | 654 | POU2AF1 | 704 | CEACAM8 | 754 | TNF | 804 | CCR3 |
| 5 | S100A12 | 55 | HLA-C | 105 | JAK2 | 155 | BCL2L1 | 205 | CSF1R | 255 | CSF2RB | 305 | PRPF38A | 355 | POU2F2 | 405 | MNX1 | 455 | GPATCH3 | 505 | ENG | 555 | ZKSCAN5 | 605 | CD180 | 655 | KLRC2 | 705 | GZMK | 755 | NOD1 | 805 | CXCL14 |
| 6 | PPBP | 56 | CXCR4 | 106 | TLR1 | 156 | SDHA | 206 | FCER2 | 256 | ROPN1 | 306 | CYLD | 356 | IL4 | 406 | CAMP | 456 | MAGEA12 | 506 | SIGIRR | 556 | PPARG | 606 | CD59 | 656 | CX3CR1 | 706 | RELB | 756 | SPINK5 | 806 | ITGA5 |
| 7 | CD55 | 57 | ITGAE | 107 | ITGA2B | 157 | CLEC7A | 207 | PYCARD | 257 | IFIT2 | 307 | TIGIT | 357 | KLRD1 | 407 | TNFSF4 | 457 | CD14 | 507 | SYCP1 | 557 | IL22RA2 | 607 | CCL28 | 657 | CD274 | 707 | NFATC1 | 757 | TLR2 | 807 | CCL21 |
| 8 | ATF2 | 58 | CYBB | 108 | TNFSF13B | 158 | CXCL5 | 208 | TAPBP | 258 | CD79B | 308 | MAP3K7 | 358 | CD34 | 408 | ATG16L1 | 458 | SPN | 508 | BLNK | 558 | GPI | 608 | IFIH1 | 658 | CD44 | 708 | IRF7 | 758 | IL17RA | 808 | TMEFF2 |
| 9 | UBC | 59 | MTMR14 | 109 | HLA-E | 159 | CCL7 | 209 | TLR5 | 259 | CD276 | 309 | HAVCR2 | 359 | IL1RAPL2 | 409 | ECSIT | 459 | IL13 | 509 | NT5E | 559 | CD40 | 609 | PVR | 659 | CFP | 709 | IRF8 | 759 | IL18 | 809 | MRC1 |
| 10 | CD53 | 60 | CASP3 | 110 | IL32 | 160 | EP300; MI | 210 | CD58 | 260 | IL7R | 310 | MAGEA4 | 360 | MS4A1 | 410 | CD8A | 460 | CLEC4C | 510 | ICAM1 | 560 | CXCL3 | 610 | EIF2B4 | 660 | ARG2 | 710 | APOE | 760 | CTLA4 | 810 | ADA |
| 11 | PPIA | 61 | REL | 111 | TREM1 | 161 | IL17B | 211 | INPP5D | 261 | STAT6 | 311 | MAGEB2 | 361 | TNFSF8 | 411 | CD19 | 461 | NOTCH1 | 511 | MAP4K2 | 561 | LILRB3 | 611 | EGFR | 661 | CXCL1 | 711 | TREM2 | 761 | TNFRSF11 | 811 | CTSH |
| 12 | BLNK | 62 | F13A1 | 112 | IL2RG | 162 | C4B | 212 | SIGLEC1 | 262 | KLRF1 | 312 | IL6 | 362 | IL22 | 412 | INPP5D | 462 | ALAS1 | 512 | MICA | 562 | CXCR5 | 612 | IRGM | 662 | CREB5 | 712 | IL17RB | 762 | CXCR6 | 812 | GTF3C1 |
| 13 | CD63 | 63 | LCN2 | 113 | LRRN3 | 163 | ITGAM | 213 | SPN | 263 | KLRB1 | 313 | MRPS5 | 363 | CASP8 | 413 | STAT5B | 463 | LY9 | 513 | CXCL10 | 563 | TTK | 613 | STAT4 | 663 | FLT3 | 713 | DOCK9 | 763 | CSF2 | 813 | SYK |
| 14 | FCGR2A | 64 | CD48 | 114 | C1QBP | 164 | PLAU | 214 | TYK2 | 264 | BST2 | 314 | IL15 | 364 | TNFSF13 | 414 | IFI27 | 464 | SSX1 | 514 | IL5RA | 564 | RORA | 614 | MAP3K1 | 664 | CDH1 | 714 | HDAC3 | 764 | IKBKB | 814 | PDGFRB |
| 15 | MAVS | 65 | CXCL16 | 115 | HLA-DQB1 | 165 | PDCD1 | 215 | MR1 | 265 | IL7 | 315 | MSR1 | 365 | RUNX1 | 415 | CCL25 | 465 | IL1RAP | 515 | C8B | 565 | IL1RAP | 615 | TCF7 | 665 | TFRC | 715 | SOCS1 | 765 | FOXJ1 | 815 | SLAMF1 |
| 16 | TXNIP | 66 | PRKCE | 116 | CD1C | 166 | CD1D | 216 | FYN | 266 | JAK3 | 316 | TLK2 | 366 | AMBP | 416 | CLEC6A | 466 | CCR6 | 516 | BAGE5 | 566 | TPTE | 616 | CX3CL1 | 666 | C3 | 716 | FUT5 | 766 | CRP | 816 | TNFRSF4 |
| 17 | IFI16 | 67 | CD68 | 117 | TUBB | 167 | PIK3CD | 217 | GZMB | 267 | CR1 | 317 | FPR2 | 367 | CNOT4 | 417 | MAP3K5 | 467 | VCAM1 | 517 | MFGE8 | 567 | CD200 | 617 | C5 | 667 | TNFRSF10 | 717 | PRG2 | 767 | MASP1 | 817 | ELANE |
| 18 | CD37 | 68 | HLA-DPB1 | 118 | TNFRSF12 | 168 | FCGR2B | 218 | NCR1 | 268 | POLR2A | 318 | IL4R | 368 | CTAG1B | 418 | TLR10 | 468 | CD207 | 518 | ULBP2 | 568 | RAG1 | 618 | IL6R | 668 | TICAM1 | 718 | LRP1 | 768 | TBP | 818 | NEFL |
| 19 | PTPRC | 69 | KRAS | 119 | PDGFC | 169 | BCL6 | 219 | CTCFL | 269 | BRAF | 319 | LILRA1 | 369 | CD40LG | 419 | GUSB | 469 | IL2 | 519 | CCND3 | 569 | CTSW | 619 | CD160 | 669 | CD4 | 719 | C8A | 769 | TLR9 | 819 | RRAD |
| 20 | HLA-DRA | 70 | INPP5D | 120 | HLA-A | 170 | CCR2 | 220 | BST1 | 270 | IFIT1 | 320 | CD1E | 370 | RELA | 420 | IRF5 | 470 | TLR4 | 520 | CCL23 | 570 | CREB1 | 620 | ATG16L1 | 670 | MAVS | 720 | IL12B | 770 | KIR2DS5; | 820 | MCAM; |
| 21 | IFITM1 | 71 | IKBKE | 121 | CHIT1 | 171 | SELPLG | 221 | IL5 | 271 | CDK1 | 321 | CD83 | 371 | PTGS2 | 421 | FCGR1A | 471 | IKBKB | 521 | IL17F | 571 | MEFV | 621 | CD27 | 671 | SAP130 | 721 | CEBPB | 771 | IL3 | 821 | INPP5D |
| 22 | IFITM2 | 72 | ATM | 122 | CD163 | 172 | CTAGE1 | 222 | IL17A | 272 | TNFRSF11 | 322 | CTSG | 372 | CHUK | 422 | INPP5D | 472 | HPRT1 | 522 | CCL3 | 572 | LAMP3 | 622 | SERPING1 | 672 | CTA-109P1 | 722 | TLR7 | 772 | CD2 | 822 | NRP1 |
| 23 | CCL27 | 73 | PECAM1 | 123 | ATG12 | 173 | IL34 | 223 | CD247 | 273 | SLC11A1 | 323 | TNFSF13B | 373 | IL21 | 423 | CSF3R | 473 | SEMG1 | 523 | ITGAX | 573 | IRAK2 | 623 | BCL10 | 673 | IL12A | 723 | EBI3 | 773 | IFNB1 | 823 | IL22RA1 |
| 24 | CD74 | 74 | PSMD7 | 124 | HLA-DMA | 174 | HLA-G | 224 | INPP5D | 274 | ABCF1 | 324 | PRKCD | 374 | SLAMF6 | 424 | IL18R1 | 474 | DUSP4 | 524 | SPP1 | 574 | TLR4 | 624 | PSMB10 | 674 | C4BPA | 724 | COL3A1; N | 774 | PRM1 | 824 | CCL4L2; |
| 25 | CD99 | 75 | DPP4 | 125 | DDX58 | 175 | NFATC3 | 225 | JAK2 | 275 | GATA3 | 325 | ITGA6 | 375 | IL15RA | 425 | IL11 | 475 | IL25 | 525 | ATG16L1 | 575 | CXCL2 | 625 | AMMECR1 | 675 | SPANXB1 | 725 | INPP5D | 775 | TFE3 | 825 | ITGAL |
| 26 | CD99 | 76 | NRAS | 126 | ISG20 | 176 | CCR7 | 226 | CEACAM6 | 276 | ATG16L1 | 326 | ATG16L1 | 376 | RIPK2 | 426 | CD209 | 476 | TLR8 | 526 | TNFRSF14 | 576 | INPP5D | 626 | C9 | 676 | ATG16L1 | 726 | LCK | 776 | DHX16 | 826 | TAB1 |
| 27 | MAPK8 | 77 | MAP2K4; | 127 | PSMB9 | 177 | EOMES | 227 | ITGA2 | 277 | CCL2 | 327 | FLT3LG | 377 | SLAMF7 | 427 | CD6 | 477 | LILRA5 | 527 | C1R | 577 | TANK | 627 | CCL26 | 677 | CCR5 | 727 | GZMM | 777 | IL12RB2 | 827 | SH2B2 |
| 28 | CTSS | 78 | LAMP2 | 128 | IFNAR1 | 178 | CD164 | 228 | CFI | 278 | C1QA | 328 | SELL | 378 | IL19 | 428 | MICB | 478 | IL12RB1 | 528 | FOXP3 | 578 | CCL8 | 628 | MERTK | 678 | TRIM39 | 728 | CCL11 | 778 | HSD11B1 | 828 | NUP107 |
| 29 | MEF2C | 79 | IFNGR1 | 129 | MAPK1 | 179 | NLRP3 | 229 | CKLF-CMT | 279 | BTK | 329 | IL23A | 379 | SELE | 429 | IL6R | 479 | CXCL6 | 529 | CMKLR1 | 579 | AGK | 629 | CD33 | 679 | CD5 | 729 | C2 | 779 | IFNG | 829 | TNFRSF11 |
| 30 | APP | 80 | G6PD | 130 | HLA-DQA1 | 180 | NOL7 | 230 | ATG16L1 | 280 | CCR4 | 330 | CD53 | 380 | GZMH | 430 | IGLL1 | 480 | LILRA5 | 530 | TAP2 | 580 | IFNA17 | 630 | CCR1 | 680 | BIRC5 | 730 | IL1RL1 | 780 | MUC1 | 830 | MAP4K2 |
| 31 | NFKBIA | 81 | KLRC4-KLR | 131 | MX1 | 181 | FAS | 231 | PRF1 | 281 | IRF1 | 331 | CLEC5A | 381 | VEGFC | 431 | ATG16L1 | 481 | IRF4 | 531 | S100A7 | 581 | ELK1 | 631 | CASP10 | 681 | F2RL1 | 731 | ABCB1 | 781 | MST1R | 831 | FN1 |
| 32 | CCND3 | 82 | IL16 | 132 | BID | 182 | ATG7 | 232 | CXCR3 | 282 | TNFSF15 | 332 | HLA-DPA1 | 382 | CDKN1A | 432 | ATG16L1 | 482 | COMMD3 | 532 | INPP5D | 582 | ITGB2 | 632 | RUNX1 | 682 | MAPK11 | 732 | IL3RA | 782 | CSF3 | 832 | CHIT1 |
| 33 | AKT3 | 83 | EWSR1 | 133 | ATG5 | 183 | TNFRSF1A | 233 | ZNF143 | 283 | LAIR2 | 333 | LAMP1 | 383 | ITGB4 | 433 | ATG16L1 | 483 | CCL1 | 533 | USP9Y; TT | 583 | DMBT1 | 633 | INPP5D | 683 | IL11RA | 733 | IL21R | 783 | SLAMF6 | 833 | ICAM4 |
| 34 | CD3E | 84 | CKLF; CKL | 134 | ADORA2A | 184 | TNFAIP3 | 234 | PSMB7 | 284 | CD81 | 334 | LTK | 384 | TLR3 | 434 | TNFRSF13 | 484 | ITGAM | 534 | IFNA8 | 584 | HLA-DOB | 634 | HRAS | 684 | CXCL9 | 734 | TNFSF11 | 784 | ATG16L1 | 834 | NLRC5 |
| 35 | ANP32B | 85 | TAP1 | 135 | LYN | 185 | FCER1A | 235 | INPP5D | 285 | NFATC2 | 335 | ATG16L1 | 385 | CCL3L3; C | 435 | PIK3CG | 485 | SERPINB2 | 535 | TMUB2 | 585 | MAP2K1 | 635 | CCL13 | 685 | FOS | 735 | IFNA1 | 785 | SBNO2 | 835 | TNFRSF17 |
| 36 | HLA-DMB | 86 | ITGB3 | 136 | IL1R2 | 186 | RUNX3 | 236 | INPP5D | 286 | ICOSLG | 336 | LILRB1 | 386 | MAPK3 | 436 | BCL2 | 486 | KLRC1 | 536 | CTSL | 586 | SPO11 | 636 | CD28 | 686 | DNAJC14 | 736 | CCL18 | 786 | THBD | 836 | HAMP |
| 37 | SELL | 87 | CD47 | 137 | ZNF205 | 187 | MARCO | 237 | CSF1 | 287 | ATG16L1 | 337 | ICAM2 | 387 | CD79A | 437 | NCAM1 | 487 | PIN1 | 537 | CD200; RP | 587 | TRAF6 | 637 | SF3A3 | 687 | CD70 | 737 | ATG16L1 | 787 | EGR1 | 837 | PASD1 |
| 38 | LCP1 | 88 | IL18RAP | 138 | LTF | 188 | NRP1 | 238 | IRAK1; MI | 288 | CXCR1 | 338 | CD3G | 388 | REPS1 | 438 | AIRE | 488 | CCL17 | 538 | IGF1R | 588 | ATF1 | 638 | ATG16L1 | 688 | MS4A2 | 738 | INPP5D | 788 | CCL14; | 838 | IL27 |
| 39 | HMGB1 | 89 | LILRB2 | 139 | BAX | 189 | STAT1 | 239 | IL1R1 | 289 | AICDA | 339 | MAPK14 | 389 | PAX5 | 439 | IFNA7 | 489 | TBX21 | 539 | POU2AF1 | 589 | CFD | 639 | CCL4L2; C | 689 | IL9 | 739 | IL3RA | 789 | BTLA | 839 | MASP2 |
| 40 | ANXA1 | 90 | CD46 | 140 | VEGFA | 190 | PSEN2 | 240 | SH2D1B | 290 | TRAF2 | 340 | NCAM1 | 390 | AXL | 440 | IL1RL2 | 490 | YTHDF2 | 540 | LGALS3 | 590 | CD80 | 640 | LIF | 690 | CD9 | 740 | PSEN1 | 790 | COLEC12 | 840 | SYT17 |
| 41 | HLA-B | 91 | LTB | 141 | TAL1 | 191 | TNFSF10 | 241 | MBL2 | 291 | EGR2 | 341 | MIF | 391 | S100B | 441 | TNFSF14 | 491 | CMA1 | 541 | IL2RA | 591 | CCR9 | 641 | C1QB | 691 | PBK | 741 | TIRAP | 791 | CFB | 841 | COG7 |
| 42 | CCL5 | 92 | STAT3 | 142 | IL10RA | 192 | ITGB2 | 242 | CCL19 | 292 | STAT5B | 342 | KRAS | 392 | C3AR1 | 442 | OAS3 | 492 | EPCAM | 542 | FADD | 592 | IFNA2 | 642 | IL23R | 692 | CYFIP2 | 742 | ATG16L1 | 792 | SAA1 | 842 | SPACA3 |
| 43 | PLAUR | 93 | USP39 | 143 | INPP5D | 193 | CXCR2 | 243 | IL13RA2 | 293 | BATF | 343 | PLA2G6 | 393 | ZC3H14 | 443 | ATG16L1 | 493 | RORA | 543 | CARD11 | 593 | MAF | 643 | TLR1 | 693 | ZNF346 | 743 | SH2D1A | 793 | A2M | 843 | RORC |
| 44 | CASP1 | 94 | IL6ST | 144 | MAP2K1 | 194 | ATG16L1 | 244 | CD86 | 294 | ATG10 | 344 | CD46 | 394 | NFKB1 | 444 | MAGEA3 | 494 | C1S | 544 | PRAME | 594 | TPSB2; TP | 644 | IRAK4 | 694 | CCL24 | 744 | IFNL2 | 794 | F12 | 844 | C7 |
| 45 | CD96 | 95 | BAGE; BA | 145 | PNMA1 | 195 | MAP2K2 | 245 | TFRC | 295 | IFNL1 | 345 | ERCC3 | 395 | CT45A1 | 445 | IL13RA1 | 495 | ARG1 | 545 | CXCL12 | 595 | CD7 | 645 | RP5-1091 | 695 | ITK | 745 | CCL20 | 795 | THY1 | 845 | TFEB |
| 46 | RUNX1 | 96 | TNFRSF1B | 146 | SMAD2 | 196 | MME | 246 | TNFRSF10 | 296 | TNFRSF8 | 346 | IRF2 | 396 | DEFB1 | 446 | PLA2G1B | 496 | BLK | 546 | CD1B | 596 | MAGEC1 | 646 | CXCL11 | 696 | SYK | 746 | TNFSF12 | 796 | SH2B2 | 846 | PIK3CG |
| 47 | IFNAR2 | 97 | MYD88 | 147 | ITCH | 197 | ENTPD1 | 247 | CD36 | 297 | LTBR | 347 | JAM3 | 397 | NFATC4 | 447 | CD84 | 497 | CNOT10 | 547 | CDH5 | 597 | TNFSF18 | 647 | TRAF3 | 697 | SSX4B; SS | 747 | ABL1 | 797 | CARD9 | 847 | CC2D1B |
| 48 | JAK1 | 98 | CREBBP | 148 | IL1B | 198 | AMICA1 | 248 | TBK1 | 298 | NFKB2 | 348 | FCF1 | 398 | DPP4 | 448 | SSX4B | 498 | MAGEA1 | 548 | ZAP70 | 598 | ALCAM | 648 | CEACAM1 | 698 | PDCD1LG2 | 748 | C8G | 798 | CCL22 | | |
| 49 | LY96 | 99 | PSMB8 | 149 | DUSP6 | 199 | CD244 | 249 | TP53 | 299 | STAT2 | 349 | ICOS | 399 | ILF3 | 449 | TXK | 499 | IL2RB | 549 | CCL16 | 599 | TGFB2; TG | 649 | CD8B | 699 | FEZ1 | 749 | IL24 | 799 | ITGA4 | | |
| 50 | THBS1 | 100 | ST6GAL1 | 150 | CD3D | 200 | OSM | 250 | INPP5D | 300 | CD22; MIR | 350 | FUT7 | 400 | LILRA4 | 450 | ICAM3 | 500 | ATG16L1 | 550 | NUBP1 | 600 | IL1A | 650 | LBP | 700 | KIT | 750 | XCL2 | 800 | PELO; ITGA1 | | |
