# Supplementary material for: Immune characterization of metastatic colorectal cancer patients post reovirus administration
Source: BMC Cancer. 2020 Jun 18;20:569. doi: 10.1186/s12885-020-07038-2 (PMC7301987; doi:10.1186/s12885-020-07038-2)

Supplementary Figure 1b – Transcriptome analysis post-Reovirus administration (Genes down-regulated 0.5-fold, $p<0.05$)

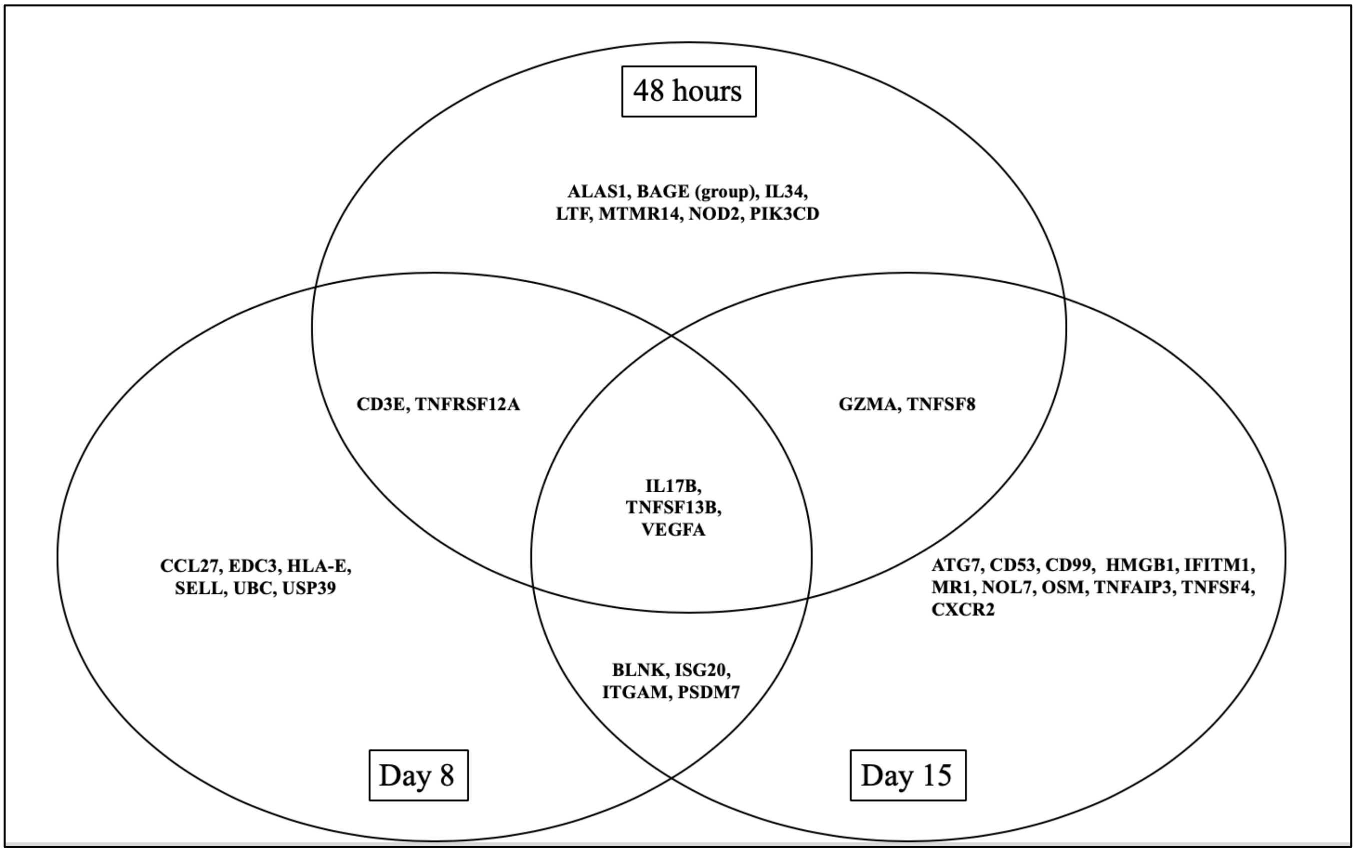

Supplement: Supplementary file 2 — Additional file 2: Supplementary Figure 1. a – Transcriptome analysis post-Reovirus administration (Genes up-regulated 2-fold, p < 0.05). b – Transcriptome analysis post-Reovirus administration (Genes down-regulated 0.5-fold, p < 0.05). [file 12885_2020_7038_MOESM2_ESM.zip › Supp Figure 1bR2.pdf]
